# Barriers to implementing health programs based on community participation: the Q method derived perspectives of healthcare professional

**DOI:** 10.1186/s12889-023-16961-5

**Published:** 2023-10-17

**Authors:** Hassan Mahmoodi, Amjad Mohamadi Bolbanabad, Abdolreza Shaghaghi, Mehdi Zokaie, Reza Ghanei Gheshlagh, Abdorrahim Afkhamzadeh

**Affiliations:** 1 Social Determinants of Health Research Center, Research Institute for Health Development, Kurdistan University of Medical Sciences, Sanandaj, Iran; 2 Department of Vice Chancellor for Health Affairs, Health Education and Promotion Group, Kurdistan University of Medical Sciences, Sanandaj, Iran; 3 Department of Health Education and Promotion, Faculty of Health, Tabriz University of Medical Sciences, Tabriz, Iran; 4 Department of Vice Chancellor for Health Affairs, Population, Family and School Health Group, Kurdistan University of Medical Sciences, Sanandaj, Iran

**Keywords:** Barriers, Health program, Community, Participation, Q methodology

## Abstract

**Background:**

Community participation in implementing health programs leads to positive organizational, social and individual consequences. This study aimed to investigate the prospects of a sample of Iranian healthcare professionals about their perceived barriers to implementing health programs based on community participation.

**Methods:**

This was a cross-sectional study that employed a Q-methodology approach. Twenty health professional sorted the 47 statements into a 9-column Q-sort diagram ranging from − 4 as not important to + 4 as very important. The data were analyzed with PQMethod 2.35 software. The centroid factor analysis and varimax rotation were used for data analysis. Factors identified were interpreted and described in terms of the participants perspectives on the phenomenon.

**Results:**

Analysis of the study participants’ viewpoints (the Q-sorts) resulted in a five factor solution (accounted for 57% of the total variance) to endorse the main barriers to the implementation of health programs based on community participation in Iran. Barriers related to government, health programs, lack of necessary skills amongst health professionals and weak coordination between departments, barriers related to community, lack of understanding of goals, benefits and transparency of roles and responsibilities were among the important emanated factors to implementing health programs based community participation.

**Conclusion:**

Health policymakers and managers should consider the five mentioned identified barriers based on the community when planning and implementing the health program participation and try to empower the community to implement the programs in Iran.

## Background

The concept of social participation and community empowerment is derived from the World Health Organization (WHO)’s strategy of health for all by 20,000 and the Ottawa charter for health promotion [1, 2] which was approved by the countries of the world at the Alma-Ata declaration in 1978 [3] and in the subsequent Jakarta declaration of the WHO focused on increasing the capacity and community empowerment, and community participation was placed as one of the most important principles of primary health care, and it was emphasized that community members should be actively involved in diagnosis, making decisions and solutions, implementation, and evaluation of health problems [4]. In addition, community participation in the planning, implementing, and monitoring of health programs is critical to prevent community disagreement, and assurance agreement on health services, which would contribute to the 17 Sustainable Development Goals (SDGs) by 2030 [5]. The WHO believes that participation in health is a type of cooperation in which people voluntarily or because of encouragement and justifications accept to interact with health-related interventions and gain benefits by providing labor or other needed resources. On the other hand, participation is also considered as empowerment through which the local community learns responsibility, diagnosis and work to solve their health problems and strives for the development of their community [6].

The concept of participation in health basically originates from the people-centered approach in development measures, and it brings to mind that in order to promote health, one should be committed to the principle of “with the people and not for the people” and based on this, adopt strategies that It should be based on people’s involvement in social affairs and urban health [7]. Along with the international community, the Islamic Republic of Iran accepted community participation as a principle in providing health services. Many programs and projects such as “Health Liaison Plan”, “Health Ambassadors Training Plan” for the implementation of the national self-care program, “Polio Eradication Mobilization Plan”, “Village Environment Improvement Mobilization Plan”, and “Country Health Benefactors Assembly” for Increasing community participation has been implemented in Iran [8, 9].

The results of some studies show that these plans have effects such as increasing the awareness and health literacy of the community, increasing the commitment of the community in participating in self-care programs, increasing the number of people visiting health centers, and increasing community participation in the construction and equipping of health centers [8, 10]. Also, community participation in implementing health programs leads to positive organizational, social and individual consequences [11].

The results of some studies in the world show that the implementation of health programs based on community participation, increase the benefit or demand for health care and treatment, and increase community awareness [12, 13]. Also, it decreases some challenges and barriers that this community was faced including the instability of financial, human and informational resources, and the low participation of some local communities [14, 15]. On the other hand, a review of literature in developed countries shows that the barriers of implementation of health programs based on community participation are related to factors such as the context of that community, infrastructural and processes factors [16].

Q-methodology is one way to identify barriers regarding health promotion programs through perspective of healthcare professional [17]. Q methodology combines and benefits from qualitative and quantitative research methods to systematically explore and describe the range of viewpoints about a topic and is therefore regarded a ‘mixed method or a ‘qualiquantological method’ [18]. Q methodology allows researchers to identify the underlying factors or dimensions that shape people’s views and attitudes, and to explore how these factors relate to each other. This can lead to a more nuanced understanding of complex issues, as well as the identification of patterns and commonalities across different groups or individuals [19]. Overall, Q methodology offers a unique and valuable approach to studying subjective experiences and perceptions, and has the potential to generate insights that may not be captured by more traditional research methods [20]. Studies show the increasing use of Q methodology in health care research [21, 22].

Thus, regarding the importance of the topic and the role of people’s participation in the implementation of the health program in Iran, especially in households, house health centers and comprehensive health service centers, the challenges of community participation in Iran have not yet been evaluated from the perspectives of health professionals, so The purpose of this study is to identify challenges from the perspectives of health professionals using a combined method (Q Methodology).

## Method

This cross-sectional study was conducted using the Q methodology during the following 6 steps using Berry and Props method [23] during the following steps.

### First and second stage: defining the discourse space

At this stage, the discourse space was formed by defining the topic or study idea. The views presented about the topic raised for the discourse space can be formed from a review of the texts and experts in this field [23]. In this study, the topic and idea raised for the discourse space was to identify challenges and strategies to improve the level of responsiveness of the health system in Iran. The discourse space included a collection of diverse materials related to the research topic, which were discussed among the people of the discourse. The people of discourse were the people whose mentalities regarding the research topic were identified by using the Q method [24]. In this study, the interviewees (sample of people) included health professionals in the Health Vice-Chancellor of Kurdistan University of Medical Sciences, who had previous knowledge about the challenges and strategies to improve the level of responsiveness of the health system in Iran.

At this stage, the health professionals who were present in the discourse (interviews) were selected as a sample of people to participate in the Q study, sorting stage. The sampling was purposeful and that the participants are selected based on having a special relationship with the subject of study (community participation) or having prior knowledge about it. The number of sample people in Q method to sort the phrases should be less than the number of phrases around the subject under study (typically 1 participant for every 3–5 statements) [20, 25]. In this study, based on final 47 phrases, the number of participants to sort the challenges and strategies to improve the responsiveness of the health system was 20 people.

### The third step: screening and selecting phrases (Q-sample)

In this study, we used the semi-structured interview. The guide to the topics of the interviews were:

1. Can you describe your experience with implementing health programs based on community participation?
2. What do you think are the main barriers to implementing such programs?
3. How do you address or overcome these barriers?
4. Can you provide examples of successful community-based health programs that you have implemented?
5. How do you involve community members in the planning and implementation of health programs?
6. How do you ensure that the health programs meet the needs and preferences of the community?
7. How do you measure the effectiveness of community-based health programs?
8. Have you encountered any challenges or resistance from community members in implementing health programs? How did you address them?
9. How do you sustain community-based health programs over time?
10. What advice would you give to others who are interested in implementing health programs based on community participation?

The general goal of the semi-structured interview is to gather systematic information about a set of central topics, while also allowing some exploration when new issues or topics emerge [26]. During semi-structured interviews with 20 experts in the health department, 67 statements regarding the challenges and strategies to improve the responsiveness of the health system were extracted. For choosing statements, we considered the following: Relevance: The statements should be relevant to the topic being studied and reflect the range of opinions and perspectives on the topic. Diversity: The statements should cover a range of viewpoints and opinions, including those that may be less commonly expressed or controversial. Clarity: The statements should be clear and concise, avoiding jargon or technical language that may be difficult for participants to understand. Balance: The statements should be balanced in terms of positive and negative views, and avoid bias towards any particular viewpoint. Pilot testing: It is important to pilot test the statements with a small group of participants before using them in a larger study, to ensure that they are understood and interpreted as intended [22, 27]. According to the steps mentioned, 47 phrases were selected.

### The fourth step: Q-sort

At this stage, the normal distribution table was designed offline in the form of Likert scale + 4 to -4. The software was provided to experts. Guidance on how to distribute expressions on the normal distribution table was provided. So that in the first stage, the purpose of the study is the number of statements selected through interviews, in the second stage, placing the phrases in three columns: I agree, I have no opinion and I disagree, in the third stage, the distribution of the statements (compulsory) in the normal distribution diagram of the Likert spectrum (+ 4 to -4), to explain the reason for choosing the phrases at both ends of the Likert spectrum from their point of view, and in the last step, to enter demographic information. Therefore, in Q, the sorting process is subjective [23], in other words, the operation of sorting items in the normal distribution network allows each participant to present his or her internal perspective through his or her sorting.

### Step five and six: analysis and interpretation of factors

The data obtained from sorting Q by the students were entered into the PQ-Method software version 2.35. The process of analysis and interpretation was done in three stages: (a) identification of factors (b) conversion of factors into factor arrays (c) interpretation of factors using factor arrays.

A) Factor Identification: Extraction of factors in PQ-Method software was done through the following sequential steps: (a) principal component analysis, (b) identification of hidden factors, (c) varimax rotation and assessment of factor loadings for eigenvalues. Above 1.00, d) estimation of the percentage of variance explained by the identified factors and (e) differentiation of interpretable factors with at least two types of correlated Q [27].
B) Converting factors into a factorial array.

The observed correlation between each of the Q rankings and an identified factor indicates the degree of opposition between the Q rankings and the identified factors [23, 28]. The manual marking mechanism in the PQ-Method software was applied for this study and correlation coefficients of at least 0.297 were considered as the cut-off point (the absolute value of the factor loading is greater than (1/96)/(√n), then the factor loading respectively, was significant with 95% confidence, that is, the value of n was equal to the number of phrases in the Q study (n = 47). were ordered for the identified factors [29]. The orders specified on a factor are used to create the factor array for that factor. The factor array represents the order of that factor (point of view) and using z-scores is determined. In fact, the array of factors determined for each factor at what level of the spectrum each expression is; therefore, according to the position of each expression, a more accurate interpretation of each factor (mentality) was achieved. P values (less than 0.05 in vs. 0.01) to distinguish expressions is also determined from the z-score [30].

C) Factorial interpretation using factorial arrays.

Distinct expressions of Q were identified and factors were interpreted contextually. The defining phrases for a factor were those with rank values of “+4”, “+3”, “-4”, “-3” in factor arrays with different scores (P < 0.05) in a given factor. Compared to their scores in other factors, the post-P-set interview was also conducted at the end of the Q-sort to confirm the recognition and interpretation of the factors of the subgroups of items among the identified factors.

## Results

The mean (standard deviation) of the age of the participants in this study was 44.90 (4.81). 60% of the participants in the study were women. 45% of the participant had public health degree. (Table 1). The average duration of the participants in the distribution of items in Q sorting was equal to 50 min. Five factors: the first factor (28%), the second factor (8%), the third factor (8%), the fourth factor (7%) and the fifth factor (6%) were identified from the point of view of health professionals, which accounted for 57% of the variance explained.

**Table 1:** Participant characteristics

Variables	Frequency (%)
Gender	
Men	8 (40)
Women	12 (60)
**Study field**	
Environment health	2 (10)
Occupational health	2 (10)
General practitioner	1 (5)
Family health	4 (20)
Public health	9 (45)
Health education	2 (10)
**Education level**	
Bachelor’s	11(55)
Master’s	8 (40)
Doctoral	1 (5)
**Duration of employment**	
Less than 10 years	3(15)
10 to 20 years	9(45)
Up to 20 years	8(40)

The rotated matrix of the factors showed that 18 participants were significantly loaded on the first factor and 5 on the second factor, 7 on the third factor, 5 on the fourth factor and 5 on the fifth factor.

After determining the scores of the factors in the rotated matrix, to form a Q table for each factor and give a score to each of the Q options, based on the calculated scores, the factor arrays were calculated and the Q options were ranked in order of importance was determined for each factor (Table 2).

**Table 2:** The Q-set statements and factor arrays in the study of barriers implement to health education programs

Items	Statements	Factors				
		1	2	3	4	5
1	Lack of community understanding toward the goals and benefits of collaborative work	1-	4-*	2-	1**	4**
2	Lack of transparency of roles and responsibilities in the planning, implementation and evaluation of the health care system	0	3-**	0	2**	4**
3	Lack of commitment to community participation, including unwillingness to share power in health-related decisions	0	1-	0	1	1
4	Reduction of social capital	2**	1-	1-	2-	2-
5	Lack of time for community members	3-	3-	3-	1**	1-**
6	Lack of skills and knowledge of community members	2-*	1-	0	0	1
7	Top-down planning	2	1*	0	1-	3
8	Lack of funds and resources and sustainability of cooperative programs	3**	1*	3*	1-	1-
9	Failure to support the presence of community members in health-based collaborative work	1	2	0	0	1
10	Lack of suitable places for community members to participate in collaborative programs	2-	0	1-	0	0
11	Implementation delay of health programs for participating volunteers	1-**	3-**	0	2	2
12	Lack of proper training for health professionals about working with the community	0	1	4**	1-	0
13	Lack of proper training for communities members to participate in collaborative work	0	1	2	2	0
14	Lack of clarity, transparency and misplaced expectations of society from cooperative works	0	0	1	1-	2
15	Limited timeline for building trust and achieving the breadth and depth of collaborative programs	3-	2-	2-	1**	2-
16	Lack of organizational commitment to collaborative work	1	1-	1	2-	2-
17	A history of poor community relations in collaborative health programs	1-	1-	4-**	1-	3**
18	Lack of comprehensive infrastructure to implement the community-based participatory program	3	2	1-	1	1-
19	Lack of flexibility to respond to the needs and demands of society in collaborative health work	1-	2-	3-	0	2-
20	Lack of management skills and knowledge of employees in community participation	2-	1	2	2	1-
21	Lack of community representatives in the service management committee and boards of directors of cooperative programs	0	0	1-	3**	1**
22	Lack of support policies for participatory programs in society	3**	2-	1	3-	0
23	Poor cooperation of community organizations	2	2-**	3	3-**	2
24	Lack of trust, respect and self-confidence in collaborative work	1-	1-	3**	3-**	0
25	Lack of interest in health programs	4-**	2	2	2-**	1
26	Lack of benefit for people participating in health programs	1-	0	4-**	4*	0
27	A history of failure of previous programs based on community participation	2-	2-	0	0	3-**
28	Failure to complete executive projects	1	1	1-	3-*	1
29	Lack of attractiveness of health programs	4-**	4**	0	0	2**
30	Lack of consider the needs of different groups in health programs	1	3-	1	0*	3-
31	Failure to meet expectations and keep people in health programs over time	1	2	0	3	3
32	Lack of understand the information provided	3-**	0	2	0	2
33	Dissatisfaction with the terms of participation	2-	1	2-	2-	0
34	Lack of participation of youth, elderly and minorities	0**	2	2-**	1	3-**
35	The information gap of community members about collaborative work	2-	0	2-	1-	0
36	The low level of people literacy in the community to participate in collaborative work	3-**	2*	1	4-**	0
37	Active community resistance around participating in collaborative programs	1-	0*	2-	3	3
38	People’s lack of trust in the government and the government system in collaborative work	4	4	2	3	1*
39	Lack of proper culturalization and preparing the necessary cultural platform for the implementation of collaborative works	0	0	2**	4**	3-**
40	Improper planning for the implementation of community cooperative works	1	2-	1	2-	1-
41	load of programs and work parallel in collaborative programs	0	1-	1	4	2
42	Existence of social and economic problems in the community	4**	3**	3-	2-*	4-
43	Existence of psychological problems in society	2**	3**	3-*	4-	4-
44	Lack of sense of duty towards solving problems in society	2	4-*	3	2	2-*
45	Lack of teamwork spirit in society	1	0	1-	0	1-
46	Poor interdepartmental coordination	2	3	4**	1-	1-
47	Lack of incentive levers to sustain and long-term public participation	3*	3*	1-	1**	2-

Asterisk (*) Indicates Significance at P < 0.05; Asterisk (**) Indicates Significance at P < 0.01)

### Factor 1: barriers related to government systems

In total 18 participants were significantly loaded on the first factor and explained a total of 28% of the variance. These participants believed that the lack of public’s trust in the government and the governmental system in cooperative works (4+), the existence of social and economic problems in society (4**), the lack of policies to support cooperative works (3**), and the lack of funds and resources and the unsustainability of participatory programs (3**) are among the main factors that hinder people’s participation in the implementation of health programs based on community participation.

### Factor 2: barriers related to health programs

Five participants were significantly loaded on the second factor and explained a total of 8% of the variance. In this factor, the participants believed that the lack of attractiveness of health programs (4+**), the lack of incentive levers in the implementation of health programs (3+*), the existence of psychological problems in society (3+**), the lack of public’s trust in the government and government systems (4+), and the existence of economic and social problems in society are the main barrierss to the community’s difficulties in implementing health programs based on community participation.

### Factor 3: barriers related to skills of health professionals and weak coordination between departments

Seven participants were significantly loaded on this factor and explained a total of 8% of the variance. These people have issues such as: lack of proper training for health professionals about working with the community (4+**), poor inter-departmental coordination (4++), lack of trust, respect and self-confidence in collaborative work (3+** ), the lack of sense of duty towards solving the problems in society (3+), and the lack of funds and resources and the unsustainability of participatory programs (3+*) were among the main barriers to the implementation of health programs based on community participation.

### Factor 4: barriers related to the community

Seven present of the variance of this factor was explained by 5 participants. These experts believed that the large amount of programs and parallel work in collaborative programs (4+), the lack of proper culture and preparing the necessary cultural platform for the implementation of collaborative work (4+**), the absence of community representatives in the management committee services and board of directors of collaborative programs (3+**), failure to meet expectations and keeping people in health programs over time (3+), and active community resistance around participating in collaborative programs (3+) They knew the main barriers to the implementation of the health program based on community participation.

### Factor 5: barriers related to understanding of goals, benefits and transparency of roles and responsibilities in health programs

Five health professionals were significantly loaded on this factor and explained 6% of the variance. These experts believed that the lack of a clear and common understanding of the community’s goals and benefits of collaborative work (4+**), the lack of transparency of roles and responsibilities in the planning, implementation and evaluation of the health care system (4+**), the history of weak community relations in collaborative health programs (3+**), top-down planning (3+), active community resistance around participation in collaborative programs (3+) and failure to meet expectations and keep people in They considered the length of time in health programs (3+) as one of the main barriers to implementing a health program based on community participation.

## Discussion

Community participation is critical for ensuring that health programs are effective, equitable, and responsive to the needs of the community. By involving community members in the planning and implementation of these programs, we can build stronger, healthier communities that are better equipped to address health challenges. The purpose of this study was to identify the challenges and strategies to improve the level of responsiveness of the health system in Iran from the perspective of health professionals using Q methodology. In this study, 5 factors including the problems of government systems in the implementation of health programs, problems related to health programs based on community participation, lack of necessary skills of health professionals and weak coordination between departments in the implementation of health programs, problems related to the community in the implementation of health program and lack of understanding of goals and benefits and transparency of roles and responsibilities in health programs based on community participation were identified and explained 57% of the variance. Studies shown that effective community participation is a key factor for Health Impact Assessment (HIA) to be successful in integrating health considerations non-health policies [31]. So that, community participation is generally considered a core element in HIA [32].

The findings of our study showed that the main factor for the implementation of the health program based on community participation is the problems related to government systems in the implementation of health programs, such as lack of public’s trust in the government and the government system in collaborative work, the existence of social and economic problems of the people. The lack of support policies for cooperative works, the lack of funds and resources, and the lack of sustainability of cooperative programs. In line with these results, studies have shown that trust is a prerequisite for community participation [33] and building trust or opportunities to participate at the decision making stage when planning, implementing, maintaining and utilising results especially for vulnerable groups is one of the key components for effective community participation [11, 34]. To cultivate strong relationships that build trust, public health must work through connections made with individuals and organizations, knowledge of existing relationships and networks in the community, and face-to-face meetings with communities that do not trust authorities. Building relationships requires sustained communication and long-term effort. Providing members with strategies for self-care and access to support if the work becomes overwhelming shows that their emotional labor is valued and helps build trust [35, 36]. On the other hand, building relationships and trust can be established through investing time, effort and resources to implement programs based on community participation [11]. Previous studies show that community behavior factors such as trust or opportunity to participate; Ability to participate, and willingness to participate in each activity can be driven community participation in programs [34]. When community members trust each other and institutions, they are more likely to share their ideas, concerns, and feedback. This feedback can be used to inform decision-making processes, identify solutions, and improve outcomes. Trust also facilitates the development of strong relationships between community members and institutions, which can lead to more effective partnerships and collaborations. Therefore, creation trust on the organizational level such as health system acts as the “social glue” that can hold different organizational structures together [37]. In this regard, one of the participants in this study believes that “people consider the government responsible for the implementation of programs and do not trust the government system due to the lack of transparency of programs and the honesty of the government in providing statistics.” Also, another participants said “due to the discrimination, one-sidedness and bad faith that the society has seen from government organizations and bodies in recent years, the expected non-participation is completely normal.”

Also, in line with the findings obtained in this study, other studies have shown that the lack of budget for staff and other resources (for example, running costs for volunteers) or limited time, is related to the sustainability problems of social participation projects [11].

The findings of our study showed that one of the main barriers to participation in the implementation of health programs based on community participation is the problems related to health programs, such as the lack of attractiveness of health programs and the lack of incentive levers in the implementation of health programs based on community participation. In line with these results, the study conducted by Nekoi Moghadam et al. (2017) has shown that the content of educational programs for health liaisons in Iran lacks attractiveness and application, and considering financial incentives for community members, including health volunteers, can be good incentives for continue their participation in the health-based program [38].

On the other hand, One potential problem with health programs that lack community participation is that they may not effectively address the specific needs of the community. Without input from community members, healthcare providers may not fully understand the cultural, social, and economic factors that impact health outcomes in the community. As a result, the program may not be as effective as it could be in improving health outcomes [39]. Maintaining human resources in the implementation of health programs requires the provision of welfare services, financial and spiritual supports. Some studies have shown that financial incentive is one of the main factors for the continuation of cooperation between the community and health providers in the implementation of the health program [40, 41]. In this regard, one of the participations believes that *“unfortunately, both in planning and in implementation, it is necessary to proceed in a directive manner and in line with the upstream documents, and much maneuvering power is not given to the environmental levels, let alone to comment and consider reward”.* Also, another person said: *“people’s participation in health programs needs to continue for a long time to reach the goal, if there is no incentive tool, people will lose interest.”*

According to the findings, other barriers to the implementation of health programs based on community participation was the lack of essential skills of health professionals and weak inter-sectoral coordination in the implementation of the health program. In line with these results, studies have also shown that the lack of training of health professionals and the community is one of the barriers to the implementation of health programs in the community [16]. Appropriate training in community participation and co-production is needed for health workers involved in the implementation of health programs. The lack of these general and specific skills is considered as a barrier to effective community participation. There is evidence from studies that training and capacity building for all sectors of society is an essential facilitator for effective community participation. All these studies emphasize the need for training and/or capacity building of different types, for different areas, and for different reasons or outcomes [42–44]. On the other hand, studies show that weak inter-departmental communication in the implementation of the health program and the participation of health volunteers are barriers to the implementation of the health program [38]. In this regard, one of the participants said: *“coherent and organized planning and activities do not exist in practice for inter-departmental participation by superior institutions. Although sometimes in different parts of the government, the issue of inter-departmental participation in the form of the instructions and directives are well seen, but they are not implemented in the executive and operational aspects or are done incompletely.”*

Our study showed that another important barrier to the implementation of a health program based on community participation is the problems related to the community including the large volume of programs and parallel work in collaborative programs, the lack of proper culture and preparing the necessary cultural platform for the implementation of collaborative work. In this regard, studies have also shown that one of the problems related to the implementation of the health program is the lack of awareness of the linguistic and cultural issues of that community, the lack of awareness of the opportunities, rights and structures in the community and the absence of community representatives in the planning of participation in the program is sanitary [16]. It seems that participatory approaches and positive outcomes, including community empowerment and health improvement, do not occur in a linear progression, but involve complex processes that are influenced by a set of social and cultural factors. A review study has shown that empowering the community and their participation in the implementation of the health program can be done at the individual, social and organizational levels [11]. In line with these contents, one of the participants believes that *“in order to create a sense of duty to solve problems, it is necessary to create a proper culture starting from elementary schools and be considered as a value throughout the entire period of education and life.”*

The lack of understanding of the benefits and goals of the health program in the community is another main barrier to the implementation of a health program based on community participation. In this regard, studies have shown that a collaborative health program can include short-term and long-term benefits for that community [45]. On the other hand, familiarizing health professionals with the goals and benefits of health programs based on community participation can directly facilitate the target community’s achievement of the goals and benefits of the participation program [46]. Also, specifying the roles and responsibilities for the community members in the implementation of the health program can have an effective role in advancing the program, because the participating people have a sense of ownership in the implementation of the program. The continuous communication and cooperation of the health system with the covered community can play an effective role in building trust between the parties, and in case of lack of communication, the health system will be in charge of implementing health programs and the community will absolve itself of this responsibility.

This current study has a number of limitations. Due to the time-consuming and the need to focus on the distribution of items on the normal Likert spectrum, the participants may have less noticed the importance of placing the items. Q-methodology is an exploratory tool, which helps in providing a useful insight into the existing views or opinions in society or a group of people. It also helps in characterizing each viewpoint. Q-methodology studies are not intended to generalize a finding or determine the proportion of individuals holding a particular opinion.

## Conclusion

Health policymakers and managers should consider the five mentioned identified barriers based on the community when planning and implementing the health program participation and try to empower the community to implement the programs in Iran.

## Data Availability

The datasets generated during and/or analysed during the current study are available from the corresponding author on reasonable request.

## Abbreviations

WHO: World Health Organization
